# Contrast stress echocardiography in hypertensive heart disease

**DOI:** 10.1186/1476-7120-9-33

**Published:** 2011-11-18

**Authors:** Mai Tone Lønnebakken, Åshild E Rieck, Eva Gerdts

**Affiliations:** 1 Department of Heart Disease, Haukeland University Hospital, Bergen, Norway; 2 Institute of Medicine, University of Bergen, Bergen, Norway

**Keywords:** Hypertension, Myocardial hypoperfusion, Concentric left ventricular hypertrophy, Arterial stiffness

## Abstract

Hypertension is associated with atherosclerosis and cardiac and vascular structural and functional changes. Myocardial ischemia may arise in hypertension independent of coronary artery disease through an interaction between several pathophysiological mechanisms, including left ventricular hypertrophy, increased arterial stiffness and reduced coronary flow reserve associated with microvascular disease and endothelial dysfunction. The present case report demonstrates how contrast stress echocardiography can be used to diagnose myocardial ischemia in a hypertensive patient with angina pectoris but without significant obstructive coronary artery disease. The myocardial ischemia was due to severe resistant hypertension complicated with concentric left ventricular hypertrophy and increased arterial stiffness.

## Introduction

Hypertension is associated with atherosclerosis and cardiovascular structural and functional changes predisposing hypertensive patients to myocardial ischemia also in the absence of significant epicardial coronary artery disease through a number of pathophysiological mechanisms. Increased arterial stiffness is common in hypertension, and associated with structural changes in the left ventricle, typically concentric hypertrophy or concentric remodelling [1-3]. Increased arterial stiffness may also contribute to myocardial ischemia as a consequence of the earlier return of reflection waves in stiff arteries and hence a reduction in diastolic myocardial perfusion pressure [4]. The combination of muscle-capillary mismatch due to concentric left ventricular hypertrophy and reduced diastolic myocardial perfusion pressure due to increased arterial stiffness may lead to severe myocardial ischemia. Typically these patients develop myocardial fibrosis with subsequent deterioration of diastolic and systolic function and finally clinical heart failure [5]. In addition, coronary microvascular dysfunction is common in hypertension and contributes to left ventricular ischemia [6]. Recently the diagnostic and prognostic value of Doppler derived coronary flow reserve measurement by vasodilator stress echocardiography was demonstrated [7]. However, myocardial hypoperfusion may also be directly visualized by contrast stress echocardiography [8]. This case report demonstrates the usefulness of including contrast stress echocardiography in multimodality cardiovascular imaging in diagnostic work-up of a patient with refractory hypertension presenting with exercise induced chest pain.

## Case Presentation

A 42-years old African man was referred for stress echocardiography due to exercise induced chest pain. Clinically he had resistant hypertension based on sitting blood pressure (140/100 mmHg) in spite of treatment with 4 different antihypertensive drugs, including a β-blocker, calcium-blocker, diuretic and angiotensin-2 blocker [9]. Twenty-four hour ambulatory blood pressure was on average 127/85 mmHg, but 39% of systolic and 64% of diastolic measurements were elevated, and the diurnal variation was reduced. Further diagnostic work-up did not reveal any signs of secondary hypertension. His medical history included complete recovery after coiling of a cerebral aneurysm associated with subarachnoid bleeding in 2004. There was a family history of hypertension and the patient had undergone coronary angiography in 2006 which was reported as normal.

**Figure 1:**
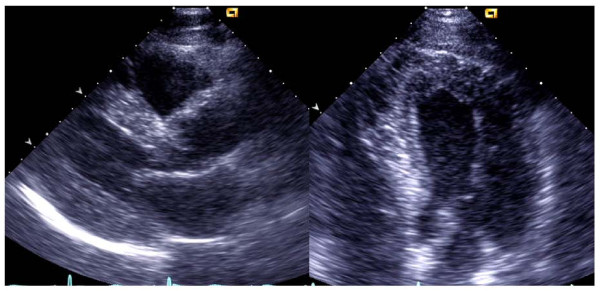
**Echocardiography demonstrating concentric left ventricular hypertrophy in parasternal long-axis and apical 4-chamber view**.

The electrocardiogram was consistent with left ventricular hypertrophy by Sokolow-Lyon criteria. The renal function was slightly reduced with elevated serum creatinine level (112 μmol/l, normal reference range 60-105 μmol/l) but normal estimated glomerular filtration rate >60 ml/min/1.73 m^2^. No micro albuminuria was present (albumin/creatinine ratio 1.2 mg/mmol, normal reference < 2.5 mg/mmol). Echocardiography demonstrated concentric left ventricular hypertrophy with left ventricular mass index 215 g/m^2 ^(normal reference value in men <116 g/m^2^) and relative wall thickness 0.52 (normal reference value <0.43) (Figure 1). Left ventricular ejection fraction was normal, but longitudinal function was reduced, consistent with reduced systolic left ventricular myocardial function and diastolic dysfunction grade 1 with normal filling pressure was also found. Left ventricular end-diastolic pressure and peak systolic left ventricular wall stress was in high normal range, 12 mmHg and 440 kdynes/cm^2 ^(normal range 275-451 kdynes/cm^2^), respectively [10-12]

**Figure 2:**
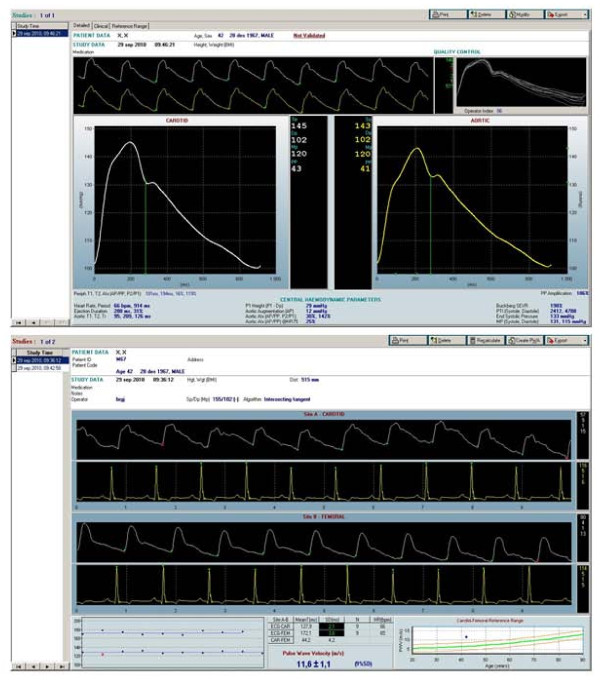
**Carotid and aortic pulse wave analysis and carotid-femoral pulse wave velocity assessed by SphygmoCor^®^**.

Pulse wave analysis by aplanation tonometry (SphygmoCor^®^, AtCor Medical Pty. Ltd, Sydney, Australia), of carotid and femoral arterial pressure waves demonstrated that central aortic systolic blood pressure and pulse pressure were in the normal range on current medication (143 mmHg and 41 mmHg, respectively) (Figure 2) [13]. The augmentation pressure was elevated (12 mmHg) and the augmentation index was in the upper normal range (12 mmHg or 25% when adjusted for heart rate). Both ejection duration (31%) and sub-endocardial viability ratio (198%) were normal. However, age adjusted pulse wave velocity was high (11.6 ± 1.1 m/s) consistent with increased arterial stiffness (Figure 2) [2,14].

**Figure 3:**
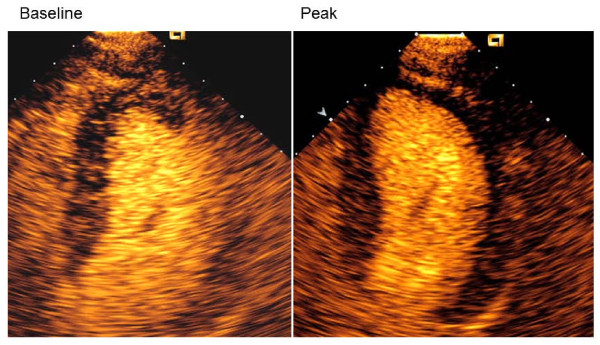
**Contrast echocardiography in apical 4-chamber view at rest and during peak dobutamine-atropine stress demonstrating delayed contrast enhancement in left ventricular myocardium**.

**Figure 4:**
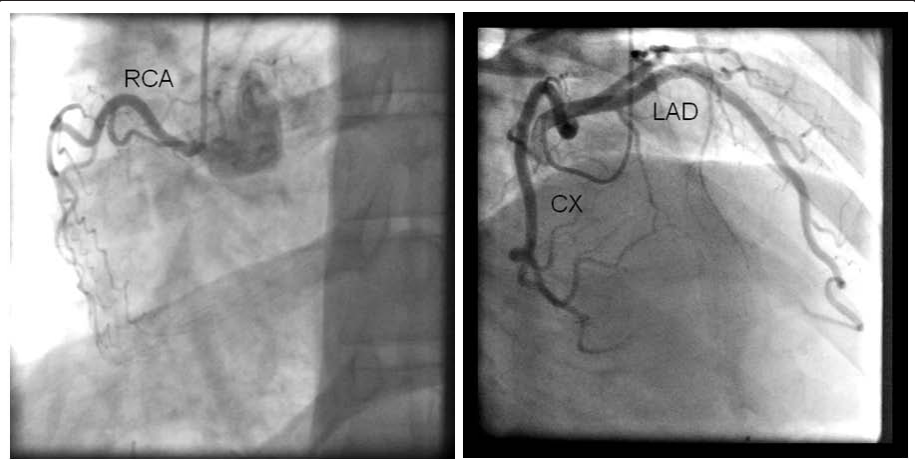
**Coronary angiography demonstrating left dominant coronary artery circulation and a hypoplastic right coronary artery, both without significant epicardial coronary artery stenoses**.

Contrast stress echocardiography with real-time low-mechanical index imaging and destruction replenishment, using Luminity^® ^(Lantheus Medical Imaging, North Billerica, Massachusetts, USA) ultrasound contrast agent and a standardized dobutamine atropine stress protocol, was performed. At rest wall motion score in all 17-segments of the left ventricle was normal, while globally delayed contrast enhancement in the left ventricular myocardium was found, in particular in the apical septum (Figure 3, Additional file 1). During dobutamine-atropine stress the patient experienced chest pain. No stress induced wall motion abnormalities were detected. However, the contrast enhancement was further delayed during stress, in particularly in the apical septum and anterior wall (Figure 3, Additional file 2). Awaiting further examination with coronary angiography he was hospitalized with acute chest pain without electrocardiographic changes, but slightly elevated serum troponin T level (18-19 mmol/l, reference value <15 mmol/l) without typical rise and fall pattern. Coronary angiography demonstrated non-obstructive coronary artery disease with non-significant changes in the left anterior descending coronary artery (Figure 4).

## Discussion

This case report demonstrates how contrast stress echocardiography may be used in cardiovascular multimodality imaging work-up of hypertensive patients with chest pain and non-obstructive coronary artery disease. The present patient had symptomatic severe myocardial ischemia documented by contrast echocardiography in the absence of significant angiographic coronary artery disease [15]. Myocardial ischemia was induced through an interaction between non-obstructive coronary artery disease, increased arterial stiffness and concentric left ventricular hypertrophy, three well known types of cardiovascular end-organ damage in hypertension [9].

Hypertension is recognized as a risk factor for development of atherosclerosis including vascular smooth muscle growth and lengthening of arteries (depicted as tortuous coronary arteries in Figure 4) and endothelial dysfunction [16]. Non-obstructive coronary artery disease is not uncommon in hypertensive patients undergoing coronary angiography due to chest pain, and detection of reduced Doppler derived coronary flow reserve during vasodilator stress echocardiography, reflecting microvascular disease and endothelial dysfunction in such patients, has been shown to predict worse prognosis in spite of normal wall motion score during conventional stress echocardiography [7,17].

Increased arterial stiffness is a well-known marker of cardiovascular risk and disease in hypertension [4]. Current guidelines recommend assessment of arterial stiffness from carotid-femoral artery pulse wave velocity, and this was high for age in this patient consistent with increased arterial stiffness [9]. The increased stiffness causes refection waves to return earlier to proximal aorta and thereby reduce the myocardial diastolic perfusion pressure. The myocardial diastolic perfusion pressure is of crucial importance to the perfusion of the myocardium, particularly during exercise, due to increased heart rate and hence shortening of the diastole.

Concentric left ventricular hypertrophy is the prognostically most unfavourable geometric pattern found in hypertension and particularly common in hypertensive patients of African origin [18]. Hypertension increases the left ventricular workload and thereby the oxygen demands of the myocardium. In particular concentric hypertrophy is also associated with a muscle-capillary mismatch as well as high endocardial wall stress which may contribute to the diagnosed myocardial ischemia in our patient [5]. Chronic ischemia may promote structural changes in the myocardium and development of fibrosis that together with functional myocardial impairment due to ischemia induced metabolic changes causes heart failure, the end stage of hypertensive heart disease.

Contrast stress echocardiography is particularly suited to detect myocardial ischemia. In accordance with current guidelines a contrast agent should be added during stress echocardiography when 2 or more left ventricular segments cannot be sufficiently evaluated [19,20]. This does not only improve wall motion assessment [20] but allows simultaneous evaluation of myocardial ischemia as contrast microbubbles are isolated intravascular tracers directly reflecting myocardial perfusion and capillary density [20]. Even if direct quantification of myocardial perfusion has proven difficult so far, qualitative detection of delayed contrast enhancement has been shown to be more sensitive to detect coronary artery disease than wall motion alone [21]. As ultrasound contrast often is indicated due to image quality, simultaneous assessment of myocardial perfusion adds important information on regional myocardial ischemia with high spatial resolution without extra cost. Furthermore, the safety of contrast echocardiography and potential side effects are well documented [20].

Also in hypertensive patients, development of wall motion abnormalities during stress echocardiography is associated with increased cardiovascular event rate [22]. Hypertension is associated with both increased prevalence of severe coronary artery disease as well as increased prevalence of non-obstructive ischemic heart disease. Echocardiographic diagnosis of microvascular disease should not be made unless obstructive coronary artery disease has been ruled out by coronary angiography or coronary computer tomographic angiography [22,23]. Diagnosis of non-obstructive ischemic heart disease is challenging and requires use of multimodality imaging as demonstrated by the present case. In particular vasodilation stress echocardiography assessing coronary flow reserve and myocardial perfusion assessment by contrast stress echocardiography or magnetic resonance imaging have proven useful in diagnosis of non-obstructive ischemic heart disease, while this diagnosis may often be missed on conventional stress echocardiography assessing only wall motion abnormality [7,22,23]. Coronary flow reserve <1.91 during adenosine stress has been associated with increased cardiovascular risk in hypertensive patients independent of the presence of wall motion abnormalities [7]. Furthermore, a coronary computer tomographic angiography study recently documented reduced prognosis in women with non-obstructive ischemic heart disease [24]. Therefore, diagnosing symptomatic myocardial ischemia in hypertensive patients without significant coronary artery disease has important clinical implications and should initiate aggressive anti-hypertensive and anti-ischemic treatment as well as risk factor modification to improve symptoms and prognosis. In particular, performing 24 hour ambulatory blood pressure recording for optimal assessment of blood pressure is important in resistant hypertension. Improved blood pressure control in resistant hypertension may be obtained by adding a small dosage of an aldosterone blocker or amiloride, the latter particularly effective in hypertension in Africans [9]. Furthermore, treatment with angiotensin converting enzyme inhibitors or statins improves microvascular function and symptoms in non-obstructive ischemic heart disease [25].

## Conclusion

Chronic uncontrolled hypertension is associated with development of cardiovascular complications including atherosclerosis, left ventricular hypertrophy, arterial stiffening and microvascular dysfunction. This can cause symptomatic myocardial ischemia even in the absence of significant epicardial coronary artery stenoses. In hypertensive patients with exercise induced chest pain and non-obstructive coronary artery disease, additional multimodality imaging including contrast stress echocardiography and assessment of vascular function is useful to identify the cause of symptoms and optimally manage such patients.

## Consent

Written informed consent was obtained from the patient for publication of this case report and accompanying images.

## Competing interests

The authors declare that they have no competing interests.

## Authors' contributions

MTL has performed the contrast stress echocardiography and drafted the manuscript. ÅER and EG have critically revised the manuscript. All authors read and approved the final manuscript.

## Supplementary Material

Additional file 1 **Contrast echocardiography at baseline demonstrating the delayed contrast enhancement, in particularly in the distal part of intraventricular septum in an apical 4-chamber view**. Click here for file

Additional file 2 **Contrast echocardiography at peak dobutamine-atropine stress demonstrating the globally delayed contrast enhancement in apical 4-chamber view**. Click here for file
